# Protective effects of quercetin and hyperoside on renal fibrosis in rats with unilateral ureteral obstruction

**DOI:** 10.3892/etm.2014.1841

**Published:** 2014-07-14

**Authors:** YANG YAN, YUAN FENG, WEI LI, JIAN-PING CHE, GUANG-CHUN WANG, MIN LIU, JUN-HUA ZHENG

**Affiliations:** 1 Department of Urology, Shanghai Tenth People’s Hospital, Tongji University, Shanghai, P.R. China; 2 Department of Nephrology, Nanjing University Affiliated Drum Tower Hospital, Nanjing, Jiangsu, P.R. China

**Keywords:** quercetin, hyperoside, obstructive nephropathy, fibrosis, experimental

## Abstract

Prevention of renal fibrosis is an important therapeutic strategy in the treatment of obstructive nephropathy. The purpose of the present study was to identify whether the combination of two natural plant-derived flavanoids, quercetin and hyperoside (QH), could inhibit renal fibrosis in the model of unilateral ureteral obstruction (UUO) in rats. QH mixtures (1:1) were fed to Wistar rats, and UUO ligation was performed on all the rats with the exception of the sham group. Masson’s trichrome staining was used for interstitial fibrosis, while immunohistochemistry and western blot analysis were used to detect the expression of α-smooth muscle actin (SMA) and fibronectin (FN). In the QH group, the expression of SMA and FN was significantly lower than that in the untreated UUO group. In addition, QH administration significantly inhibited the SMA and FN expression of mesangial cells induced by interleukin-1β. Consequently, it was evident that combinational QH therapy prevented UUO-induced renal fibrosis. Based on these findings, the combinatorial intervention of phytomedicine may present an improved treatment strategy for renal fibrotic disease.

## Introduction

Obstructive nephropathy (ON) is one of the most important causes of chronic kidney disease in children and infants (1–2). Renal interstitial fibrosis is the final pathway and the major pathological basis of ON. Thus far, a number of experimental investigations, both *in vitro* and *in vivo*, support the hypothesis that numerous factors contribute to the development of renal interstitial fibrosis, including the cell itself, extra cellular matrix, cytokines, growth factors, and the interactions between these factors (3–4). Epithelial-to-mesenchymal transition (EMT) has been hypothesized to play an important role in this process in recent years (5). From an experimental point of view, renal obstruction caused by unilateral ureteral obstruction (UUO) is the most classical model of induced renal fibrosis (6).

Dietary flavonoid quercetin is known to promote optimal health, partly via its anti-oxidation effect against reactive oxygen species (7). This compound decreases oxidative stress to improve antioxidant status, inhibits liver cell apoptosis in diabetic rats and alleviates renal fibrosis in western-style diet-fed C57/BL6J mice (8). Recently, quercetin was found to regulate inflammatory gene expression in high fat diet-fed mice (9) and to possess therapeutic effects that aided the recovery of renal morphology following UUO in neonatal rats (10). Of note, it has been demonstrated that progressive tubulointerstitial and glomerular damage persisted in the obstructed and contralateral kidney, and a decrease in the glomerular filtration rate and an increase in proteinuria occurred at the end of one year following the relief of UUO (11). Consequently, novel therapy approaches are required to prevent the progression of renal injury along with surgical intervention.

Hyperoside, as a major compound of glycoside flavanols secreted in natural plants, has been extensively used for the clinical treatment of anti-oxidation and analgesia; however, it is not clear as to whether it exhibits an anti-fibrotic effect in renal scarring. Findings of a previous study conducted in our laboratory showed that a combination of quercetin and hyperoside (QH) from a traditional Chinese herb, *Abelmoschl Manihot*, showed satisfactory anti-proliferative activities in 786-O human renal cancer cell lines (12). However, no evidence exists regarding the therapeutic effects of the two compounds for renal fibrotic lesions. Therefore, the present study was carried out to detect the actions of QH on smooth muscle actin (SMA) and fibronectin (FN) expression, and to evaluate the effects of concomitant QH administration in rats with experimentally induced UUO.

## Materials and methods

### Reagents

Polyphenolics were extracted from a standardized QH dehydrate supplement (ratio 1:1) in capsule form which was provided by Suzhong Pharmaceutical Co. (Taizhou, China). Anti-FN, anti-α-SMA and anti-β-actin antibodies were purchased from Santa Cruz Biotechnology, Inc. (Santa Cruz, CA, USA). Dulbecco’s modified Eagle’s medium (DMEM)/F12 medium and fetal bovine serum were purchased from Gibco-BRL (Grand Island, NY, USA) and Perbio Science Company (New Zealand), respectively. The bicinchoninic acid protein kit and all other chemical reagents used in the experiments were purchased from Sigma-Aldrich (St. Louis, MO, USA).

### Animal experiments and western blot analysis

Male Wistar rats (body weight 230–250 g) were randomly divided into three groups. The sham group (n=6) did not receive an operation of UUO. The untreated UUO group (n=12) received an operation of UUO without treatment of QH. The UUO + QH group (n=12) received an operation of UUO and treatment of QH. The UUO groups with and without QH treatment were further divided into two time points at three and six days. The rats were fed with QH (0.1 ml/10 g) once 16 h before surgery began and 8 h after surgery, then once a day at 9:00 am for five days. All the rats were sacrificed 24 h later. The harvested kidneys of these rats were established in the laboratory based on the mechanical sieve method (13). The methods of western blot analysis have been described previously (14).

### Cell culture and indirect immunofluorescence analysis

The mesangial cells (SV40 MES 13) were cultured in DMEM/F12 medium at 37°C and 5% CO_2_, which consisted of 15% fetal calf serum and penicillin/streptomycin (100 μg/l). The cells were grown in six-well culture plates (Nunc™, Thermo Fisher Scientific, Inc., Waltham, MA, USA) with 0.2×10^6^ cells/well density. The previous medium was replaced with serum-free DMEM/F12 medium cultured for 16 h to synchronize the cells, subsequent to fusion of 80% of the cells. Different concentrations of QH (0–60 μg/ml) were added to the medium according to the test requirements at the same time. Experiments were located with the control group. Indirect immunofluorescence analysis was performed as previously described (14).

### Statistical analysis

Data were analyzed by the SigmaStat statistical software (Jandel Scientific, San Rafael, CA, USA) and SigmaPlot (SPSS, Inc., Chicago, IL, USA). P<0.05 was considered statistically significant.

## Results

### Animal model of renal fibrosis can be successfully produced by UUO

**Figure 1:**
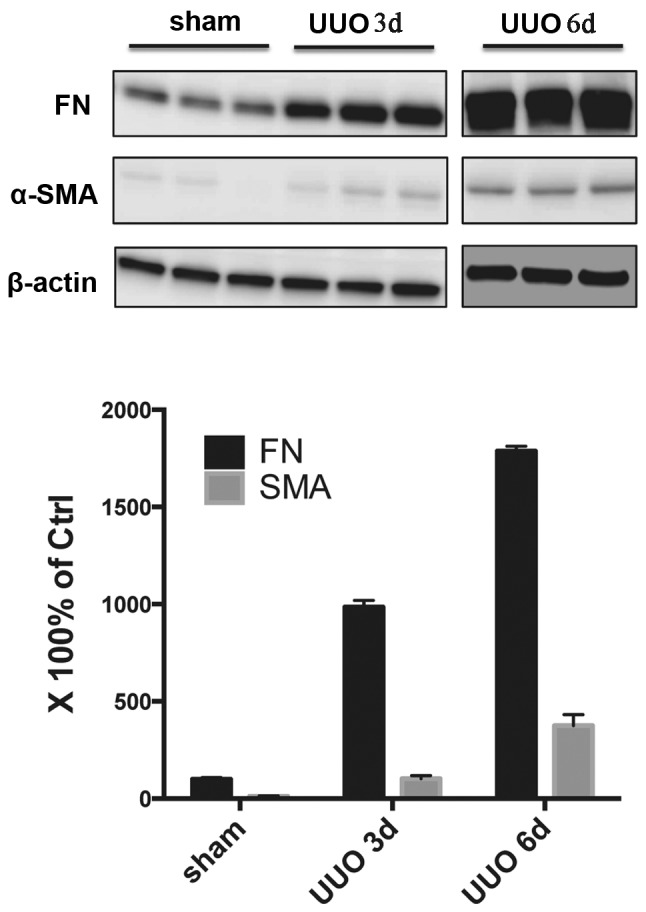
α-SMA protein and FN expression in the UUO versus the sham group. α-SMA and FN were significantly upregulated in the UUO compared with the sham group, and the expression of α-SMA was higher in the 6d compared with the 3d group (P<0.05). UUO, unilateral ureteral obstruction; SMA, smooth muscle actin; FN, fibronectin; Ctrl, control; 3d, three day; 6d, six day.

Negligible levels of α-SMA protein were detected in the sham group. However in the untreated UUO group, the expression of α-SMA was significantly increased. The expression of α-SMA was higher in the six day group compared with the three day group. In addition, a small amount of FN was expressed in the renal tissue of rats from the sham group. UUO significantly increased the expression of FN, and with the extension of obstruction time, FN expression increased significantly (Fig. 1).

### QH reduces the expression of fibrosis-related proteins in the obstructed kidney

**Figure 2:**
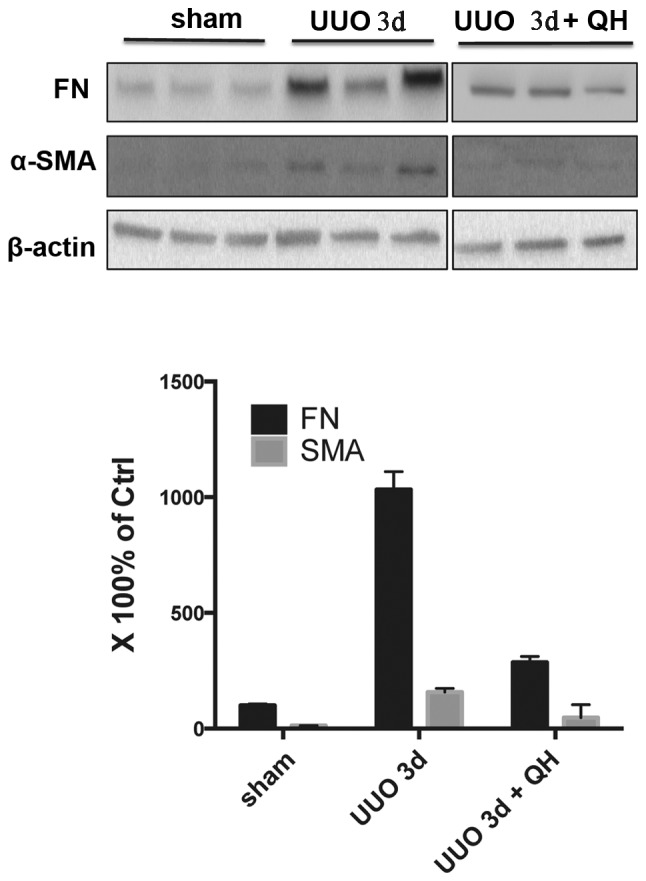
Effects of three days of QH therapy on α-SMA and FN protein expression. α-SMA and FN were significantly suppressed by three days of QH intervention compared with the untreated UUO group (P<0.05). UUO, unilateral ureteral obstruction; SMA, smooth muscle actin; FN, fibronectin; Ctrl, control; 3d, three day; QH, quercetin and hyperoside.

**Figure 3:**
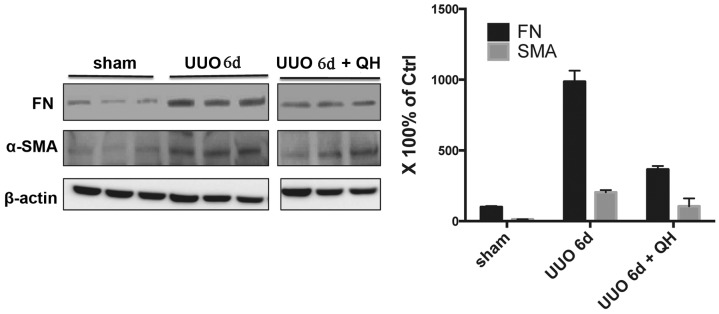
Effects of six days of QH therapy on α-SMA and FN protein expression. α-SMA and FN were significantly suppressed by six days of QH intervention compared with the untreated UUO group (P<0.05). UUO, unilateral ureteral obstruction; SMA, smooth muscle actin; FN, fibronectin; Ctrl, control; 6d, six day; QH, quercetin and hyperoside.

As shown in Fig. 2, the kidney tissue of the sham group exhibited a negligible expression of α-SMA. However, the protein expression of α-SMA in the untreated UUO group at three days was increased, which was significantly reduced by QH treatment of 0.1 mg/kg/day. Low protein levels of FN were expressed in the sham group, however, these increased almost 10-fold following UUO for three days, while the expression levels of FN decreased significantly when treated with QH compared with the untreated UUO group. Similar results were also evident in the six day group (Fig. 3).

### QH reduces the expression of fibrosis-related protein in mesangial cells in vitro

**Figure 4:**
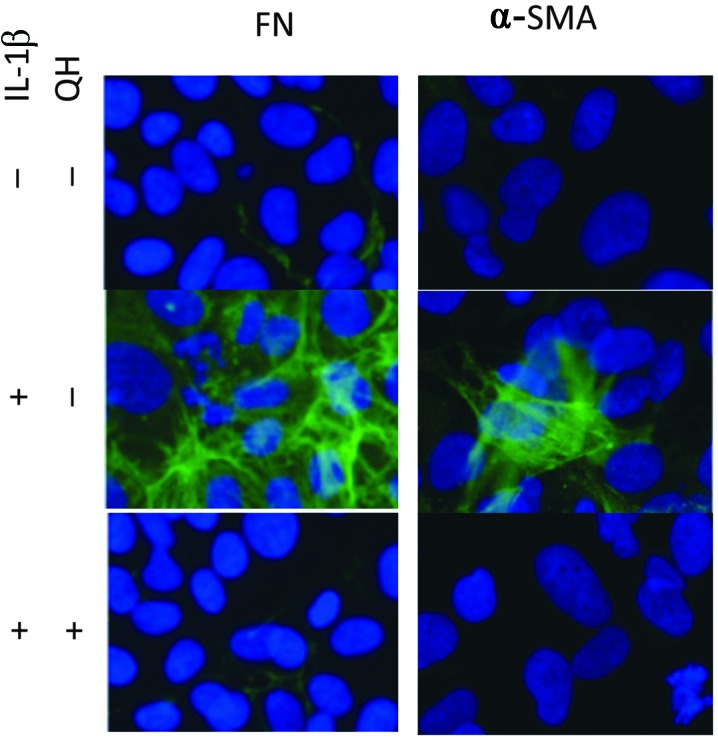
Effect of QH on expression of α-SMA and FN in mesangial cells *in vitro*. There was a low expression of FN and α-SMA in mesangial cells, while IL-1β stimulation significantly activated FN and α-SMA. However, QH intervention significantly reduced the expression levels of the two proteins. SMA, smooth muscle actin; FN, fibronectin; QH, quercetin and hyperoside; IL-1β, interleukin-1β.

The potential mechanism of QH in renal fibrosis therapy was investigated. The results showed that there was a low expression of FN and α-SMA in mesangial cells. The protein concentration of mesangial cells stimulated by interleukin-1β (IL-1β) showed a significant difference compared with the control group. However, QH was able to reduce the expression of FN and α-SMA in mesangial cells (Fig. 4).

## Discussion

The present study confirms that the combinatorial treatment of QH has the ability to reduce renal expression of α-SMA and FN in UUO rats, which are traditional models of renal fibrosis. Furthermore, the upregulation of kidney mesangial FN and α-SMA expression secondary to IL-1β induced inflammation was also suppressed by QH.

Quercetin can prevent lipid peroxidation by blocking the action of xanthene oxidase, chelating iron, and the directly scavenging hydroxyl radical (15–17). Hyperoside can prevent lipid peroxidation through iron chelation, free radical scavenging and the blockade of tyrosine kinase enzymes responsible for apoptosis in renal epithelial cells that are triggered by oxidative stress (18–19). As reported in the literature, QH could reduce renal ischemia-reperfusion injury mediated through infiltration of macrophages by interfering with inducible nitric oxide synthase activity (20).

According to a previous study, one of the key strategies for ON should be the prevention and reversal of interstitial renal fibrosis as well as relief of obstruction (21). QH provides two of these agents. To the best of our knowledge, this is the first study comparing the effects of QH on fibrogenesis in a UUO model. In addition, the association between α-SMA and FN expression and hyperoside was revealed for the first time in the present study.

Recently, a proteasome inhibitor was shown to abolish transforming growth factor-β-mediated α-SMA and FN induction, by blocking Ski-related novel protein N degradation, using human kidney proximal tubular epithelial cells. α-SMA was vital for the kidney fibrosis process. Fibrosis was associated with enhanced expression of FN in model of dyslipidemia induced renal fibrosis (22). Inhibition of FN expression significantly attenuated the expression of pro-fibrotic signals, collagen formation and the proliferation of fibroblasts (23).

Although various factors may be attributable to renal fibrosis, a definite mechanism for the initiation and progression of this complex process has not been elucidated. In the present study, the satisfactory therapeutic effects of QH were revealed by evidence-based medicine. In the present data, immunohistochemical analysis demonstrated that the expression of α-SMA and FN was inhibited by QH intervention, as well as the marked suppression observed in the *in vitro* experiment, all of which provided novel experimental evidence for the treatment of renal fibrosis. Future studies are required to determine how QH influences the proliferation of renal resident cells, its anti-inflammation mechanism, and whether such mechanisms are operative *in vivo* and *in vitro.* This will enable the development of novel interventions for the protection of humans from renal scarring for therapeutic purposes.
